# Children Residing in Low-Income Households Like a Variety of Vegetables

**DOI:** 10.3390/foods7070116

**Published:** 2018-07-20

**Authors:** Francine M. Overcash, Marla Reicks, Allison Ritter, Tashara M. Leak, Alison Swenson, Zata Vickers

**Affiliations:** 1 Department of Food Science and Nutrition, University of Minnesota-Twin Cities, 1334 Eckles Ave, St. Paul, MN 55108, USA; mreicks@umn.edu (M.R.); ritt0209@umn.edu (A.R.); tml226@cornell.edu (T.M.L.); witm0005@umn.edu (A.S.); zvickers@umn.edu (Z.V.); 2 Division of Nutritional Sciences, Cornell University, 416 Savage Hall, Ithaca, NY 14853, USA

**Keywords:** vegetables, liking, variety, children, low-income

## Abstract

Child vegetable intake falls far below the minimum recommended levels. Knowing which vegetables children may like help those responsible for providing vegetables to children to improve intake. The objective of this study was to measure vegetable liking for a wide variety of vegetables by a racially and ethnically diverse population of 9–12-year old children from low-income families. Children rated their liking of 35 vegetables using a 10-point hedonic scale. We tabulated the number of children that found each vegetable acceptable (ratings of ‘okay’ or above) and the number that found each vegetable unacceptable (ratings below ‘okay’). More than 50% of children who had tried a vegetable considered it acceptable. A large majority of the vegetables had mean ratings in the acceptable range. Corn was the most liked vegetable, closely followed by potatoes, lettuce, and carrots. Artichoke had the lowest mean liking, followed by onion and beets. We found children liked a wide variety of vegetables which offers counter evidence to the commonly held perception that children do not like vegetables.

## 1. Introduction

While vegetable intake is associated with positive health outcomes [1], less than 10% of U.S. children meet the minimum recommendations for vegetable intake [2]. Liking of vegetables is a key precursor of intake [3] as children eat what they like and avoid what they dislike [4]. Improving liking may increase intake of vegetables by children and result in health benefits.

Many researchers have claimed vegetables are the least liked food group among children with some vegetables (e.g., dark green) liked less than others [5,6,7,8,9,10]. Cooke and Wardle examined the development of food preferences in children to determine the best and least liked foods [11]. Results from 1291 4–16-year-old British children who completed a 115-item food preference questionnaire showed that 6 of the 10 least liked foods were vegetables (spinach, leeks, green squash, rutabaga, sprouts, and turnip). Patton and Carver examined preference for food groups during school lunch. They used the amount of plate waste as a measure of preference for specific food groups [12]. They found plate waste from vegetables was greater than plate waste from protein-rich foods and from fruit and fruit desserts.

Studies examining child liking of vegetables have cited bitter taste, low-calorie density, and inadequate exposure as reasons children report disliking vegetables. Humans are predisposed to reject bitter taste [13], partly as self-protection because natural poisons are bitter-tasting [14]. As a result, the bitter taste of vegetables may contribute to their rejection by children. However, Dinehart and colleagues showed that bitter taste was not a strong predictor of vegetable dislike (semi-partial correlation coefficient of liking with bitter taste = −0.22) [15]. Gibson and Wardle surveyed 416 mothers of 4–5 year olds about their children’s food likes and dislikes to determine whether energy-dense vegetables were more preferred over less energy-dense vegetables [16]. Energy density was a significant predictor of vegetable liking. Repeated exposure to vegetables has increased child liking of vegetables in many studies [8,17,18,19,20]. The “mere exposure effect” explains this increased liking [21]. The more children are exposed to vegetables, the more familiar the vegetables become, and the more liking is increased.

Hunt and colleagues surveyed 28 4th graders to determine if they had tried 15 different vegetables [18]. If they had tried the vegetable, they were asked to rate their liking of it. They then administered the same survey 2 years later. They observed that children generally accepted a variety of vegetables once they tried them. The more a vegetable was tried, the more it was liked. For example, at baseline, over 82% of the children reported having never tasted broccoli, and only 18% of children who had tasted it reported liking it “very much.” Two years later, over 80% of the children who had tried a vegetable (broccoli, cabbage, peas, lettuce, green beans, carrots, and corn) reported liking it “very much” [18].

The limited number of studies that have reported liking of individual vegetables among children have found that some are commonly liked and others are commonly disliked. Chu and colleagues measured Canadian children’s liking of specific vegetables [5]. Liking of 5 vegetables (carrots, broccoli, green beans, tomatoes, and spinach) was rated using a 3-point scale (like a lot, like a bit, don’t like) by 3398 children, aged 10–11 years. Carrots were the most liked vegetable (56% of children liked them a lot; an additional 38% liked them a bit). Spinach was the least liked vegetable (23% of children liked it a lot; 51% of children liked it a bit). Pérez and colleagues administered a survey to a sample of 3534 children and young adults (aged 2–24 years) in Spain to examine vegetable liking [7]. Participants were asked to rank their top 3 favorite vegetables out of a group of 7 vegetables (artichoke, cauliflower, spinach, asparagus, carrots, lettuce, and tomato). Tomato and lettuce were the most preferred vegetables. Corn has been reported to be one of the most familiar and most liked vegetables [18,22,23]. Brussels sprouts, spinach, and beets have been suggested as some of the least liked [11,18,24].

Children of this age group have been found to exert more independence in making food choice decisions compared to younger children [25]. Because liking is a consistent determinant of child vegetable intake [18,26,27], information on liking of specific vegetables can broaden the number of vegetables incorporated into efforts aimed at increasing variety and consumption. Parents may find the results useful for guiding their children’s vegetable choices. For example, economically-constrained parents who have been shown to base food decisions on liking in order to reduce food waste [28] may benefit from learning the variety of vegetables children like. The objective of this study was to measure liking of a wide variety of vegetables by a racially and ethnically diverse urban population of 9–12-year-old low-income children.

## 2. Materials and Methods

### 2.1. Participants

Participants were 149 children (aged 9–12 years) combined from 2 in-home intervention studies (46 from the first study; 103 from the second study) [29,30]. The largest proportion of children were Black/African American (37%) followed by Other (32%; which included Asian/Pacific Islander, Native American, Mixed Race and Hispanic ethnicity), and White (19%) (Table 1). Parent–child pairs in both studies were recruited primarily through flyer/email campaigns directed at low-income families in the Minneapolis-St. Paul metropolitan area from 2014–2016. Eligibility criteria included; (1) the child must be 9–12 years old; (2) the family must qualify for some form of public assistance and (3) the child must be able to read, speak, and understand English. The majority (62%) of children were female and aged 9–10 years (65%). The two intervention studies and the current analyses were approved as part of the same protocol by The University of Minnesota Institutional Review Board.

### 2.2. Procedure

Children rated their liking of a variety of vegetables common among the different racial and ethnic groups of the study population. The first and second study asked children to rate 36 and 37 different vegetables, respectively. In the first study, children were asked to rate their liking of 2 categories of beans: (1) black beans and (2) other beans (bean dishes, kidney, lentil, hummus), whereas in the second study they rated their liking of just 1 category of beans (black, bean dishes, kidney, lentil, hummus). In the second study, children were asked to rate 2 categories of squash: (1) squash (e.g., acorn, butternut/pumpkin) and (2) zucchini/other yellow summer squashes, whereas in the first study they rated liking of only 1 squash category (butternut, zucchini). In the second study, children rated liking of 2 categories of peppers: (1) peppers (e.g., red, orange, green) and (2) hot peppers (e.g., chilies), whereas in the first study they rated liking of 1 pepper category (red, orange, green, hot). Weighted averages were computed to combine the 2 categories of beans in the first study and the 2 categories of squash and of peppers in the second study. A total of 35 vegetables were in the final dataset (Table 2).

For both studies, iPad^®^ surveys (QuickTapSurvey^®^, www.quicktapsurvey.com, Toronto, Canada) were used to collect child liking ratings. The liking rating scale was comprised of values across a 10-point labeled hedonic scale (1—“Hate it” to 5—“It’s okay” to 10—“Love it”). This type of rating scale has been validated for testing in children [31]. The first 3 questions of the survey were example questions to help ensure the child understood how to use the slide rating scale. The slide rating scale required the child to touch a slider on the scale and move it to their rating on the scale. Staff guided each child individually through 3 example questions: “Think of a food you love”, “Think of a food you hate”, and “Think of a food that you think is just okay”. After each example question, the child moved the slider on the scale to the appropriate location. When the example questions were completed, the child proceeded with the questions about the specific vegetables.

The question “Have you ever tried (name of vegetable)” was first asked for each vegetable. If they touched ‘no’, the next screen asked about “ever trying” the next vegetable. If the child touched ‘yes’, the next screen asked the child to rate their liking of the vegetable. A two-sided handout consisting of 35 small colored pictures of each individual vegetable arranged in the same order (alphabetical) as in the survey was provided as a visual aid. Completion time for the child liking surveys ranged from about 4 to 9 min.

### 2.3. Data Analysis

Mean liking ratings, standard errors, and confidence intervals for each vegetable were calculated. The number of children that found each vegetable acceptable and unacceptable was tabulated. A rating greater than or equal to 5 indicated the child found the vegetable acceptable, whereas a rating less than 5 indicated the child found the vegetable unacceptable. The percentage of children who found the vegetable acceptable was calculated as the total number of children who found the vegetable to be acceptable divided by the total number of children who ever tried the vegetable. We used an analysis of variance (child liking was the dependent variable; vegetable was a fixed predictor and child ID was a random predictor) to determine whether the vegetables differed in liking. We used Student–Newman–Keuls tests to identify groups of vegetables that were not significantly different from each other in liking. SAS^®^ software (Statistical Analysis System, Version 9.4, 2017, Cary, NC, USA) was used to analyze the data.

## 3. Results

**Figure 1:**
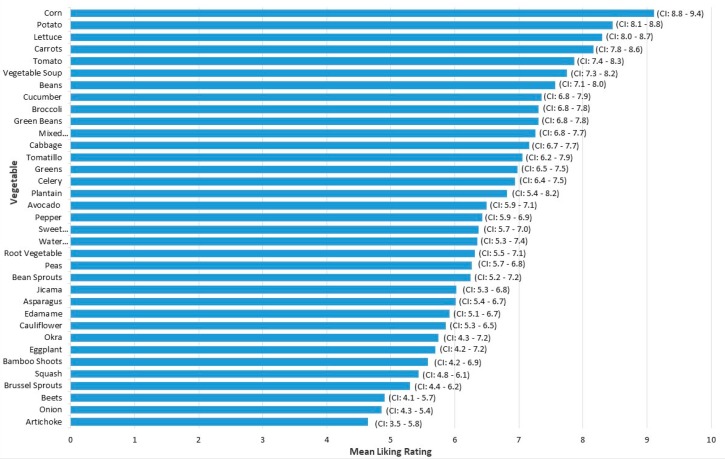
Mean liking ratings for children of 35 vegetables on a 10-point hedonic scale. Vegetables are in descending order from most liked (Corn) to least liked (Artichoke). Rating scale: 1 = Hate it, 5 = It’s okay, and 10 = Like it a lot. Standard error represented by error bars. CI is the 95% confidence interval around the mean.

Corn was the most liked vegetable with a mean liking rating of 9.1. Liking of potatoes, lettuce, and carrots closely followed liking of corn (all means ≥8.2). Artichoke had the lowest mean liking (4.6), followed by onion (4.9), and beets (4.9). These three vegetables were the only vegetables that had mean liking below 5. Mean liking across all 35 vegetables was 6.9. (Table 2, Figure 1).

**Figure 2:**
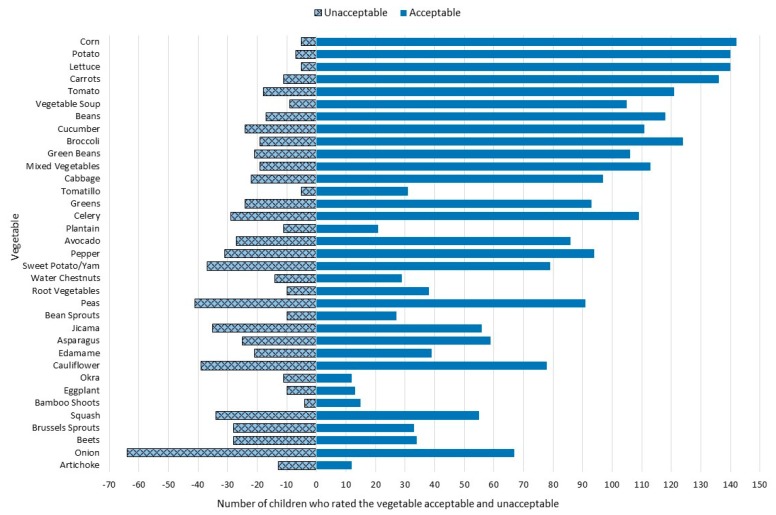
Number of children (out of 149) that found each vegetable acceptable and unacceptable. Acceptable was a rating ≥5; unacceptable was a rating <5. The vegetables are listed in order of descending mean liking ratings.

Artichoke was the only vegetable where less than 50% of children who tried it, considered it acceptable. Corn, potato, lettuce, carrots, and vegetable soup were the 5 vegetables that more than 90% of children who tried them, considered them acceptable (Table 2, Figure 2). Of the 19 children who had ever tried bamboo shoots, 79% found them acceptable. Onion and okra were two vegetables where the percentage of children who found them acceptable was almost equal to the percentage who found them unacceptable. However, onion was a much more tried vegetable among the children compared to okra (88% vs. 15% respectively) (Table 2).

## 4. Discussion

The results of this study are in agreement with previous work [18] showing that the most tried vegetables were also the most liked vegetables. In the current study, corn, potato, lettuce, carrots, and tomato were both the most liked and the most tried (more than 93% of children had tried them). Hunt and colleagues most-liked vegetables were carrots, green beans, corn, lettuce, and cabbage [18]. Carrots, green beans, and corn were also the most tried with none of their children reporting “Never tasted” them. Lettuce and cabbage were close behind with only 4% of children having checked “Never tasted”. This association between the most tried and most liked vegetables suggests that once children try a vegetable, they are willing to continue eating it at subsequent opportunities. Those subsequent opportunities would increase exposure to the vegetable, leading to greater familiarity, and in turn, improved liking as proposed by the “mere exposure” theory [8,21]. On the other hand, not all of the most tried vegetables in our study were also the most liked. The most striking example, onion, was tried by 88% of the children, yet, it was the second least liked vegetable. Onions are often found raw as a topping to popular foods like hamburgers and hot dogs. Children may not have liked the taste of raw onions and used this version of the vegetable to rate its overall liking. They may be less aware of the presence of onions in mixed dishes where their flavor and texture blend with other components.

Our most liked vegetables (e.g., carrots, corn, potato, lettuce, tomatoes) were also among the most liked vegetables in previous studies [5,7,18]. The prevalence of these vegetables in dishes across different cultures indicates wide-spread familiarity [32,33]. The exposure and evaluative conditioning that accompany familiarity are very important for determining liking [34]. With the exception of onions, most of the least liked vegetables were the least tried, suggesting that children had less exposure and less positive social conditioning to them. Carrots are considered one of the sweeter vegetables while potatoes and corn are starchy, energy-dense vegetables [35] offering an additional explanation for their well-liked status. Brussels sprouts can often taste bitter, and raw onions can be strongly pungent, likely contributing to children’s dislike. Numerous articles have reported that dark green and red/orange vegetables are the least consumed [36,37], yet the data from the current study show some of these vegetables (e.g., carrot, tomato, broccoli) were well-liked. Parents may not be aware that many children actually like these types of vegetables and therefore may not incorporate them into meals. As a result, opportunities for children to consume these vegetables may be reduced.

We found children liked a wide variety of vegetables which offers counter evidence to the commonly held perception that children do not like vegetables. This information may broaden the selection of vegetables for research interventions that promote vegetable consumption. Our results may also be of interest to parents for meal preparation at home, the setting where children consume about 2/3 of their daily calories [38]. Gaining knowledge of the variety of vegetables children have reported they like may inspire parents to prepare a greater variety of vegetables for their children. Because liking is important for new product development, the food industry may use the findings to broaden the types and amounts of vegetables formulated into their products and suggested as recipe additions, especially those developed for children.

### Limitations

A major weakness of the current study is the small number of participants compared to population-based studies that have measured liking of individual vegetables [5,7]. Therefore, generalizations drawn from our findings may be limited. Nonetheless, our participants were racially and ethnically diverse and rated a much larger number of vegetables than previous studies. The parent–child pairs in our study self-selected into their nutrition-related studies and therefore may have already had sufficient exposure to healthier foods like vegetables to elicit liking. However, Overcash [39] showed mean daily child vegetable intake among the current study population was comparable to nationally representative dietary intake data (NHANES) [40], potentially weakening this limitation. The study population was from an urban setting in the Midwest and therefore results may not be generalizable to other parts of the country.

## 5. Conclusions

Our findings show that a racially and ethnically diverse group of 9–12-year-old children residing in low-income households liked a wide variety of vegetables. Corn, lettuce, carrot, tomato, and potato were the most liked and most tried vegetables among children.

## Figures and Tables

**Table 1:** Number and percent of participants by sex, age, and race/ethnicity (*n* = 149).

Characteristic	*N* (%)
Child sex	
Female	92 (62)
Male	57 (38)
Child Age	
9	46 (31)
10	51 (34)
11	27 (18)
12	25 (17)
Child Race/Ethnicity	
White	28 (19)
Black/African American	55 (37)
Other (e.g., Asian/Pacific Islander/Native American/Hispanic)	48 (32)
Mixed Race	18 (12)
Hispanic Ethnicity	46 (31)

**Table 2:** Summary of child liking data (*n* = 149).

Vegetable	Mean ^1^ (SE)	Number of Children Who Found Vegetable Unacceptable ^2^	Number of Children Who Found Vegetable Acceptable ^3^	Number of Children Who Ever Tried Vegetable	Percent of Children Who Found Vegetable Acceptable ^4^	Mean Liking Ratings by Student–Newman–Keuls Groupings ^5^
Corn	9.1 (0.2)	5	142	147	97	A
Potato	8.5 (0.2)	7	140	147	95	AB
Lettuce	8.3 (0.2)	5	140	145	97	ABC
Carrots	8.2 (0.2)	11	136	147	93	ABC
Tomato	7.9 (0.2)	18	121	139	87	ABCD
Vegetable Soup	7.8 (0.2)	9	105	114	92	BCD
Beans	7.6 (0.2)	17	118	135	87	BCDE
Cucumber	7.4 (0.3)	24	111	135	82	BCDEF
Broccoli	7.3 (0.2)	19	124	143	87	BCDEF
Green Beans	7.3 (0.2)	21	106	127	83	BCDEF
Mixed Vegetables	7.3 (0.2)	19	113	132	86	BCDEFG
Cabbage	7.2 (0.3)	22	97	119	82	BCDEFG
Tomatillo	7.1 (0.4)	5	31	36	86	BCDEFGH
Greens	7.0 (0.3)	24	93	117	79	BCDEFGH
Celery	6.9 (0.3)	29	109	138	79	BCDEFGHI
Plantain	6.8 (0.7)	11	21	32	66	CDEFGHI
Avocado	6.5 (0.3)	27	86	113	76	DEFGHIJ
Pepper	6.4 (0.3)	31	94	125	75	DEFGHIJ
Sweet Potato/Yam	6.4 (0.3)	37	79	116	68	DEFGHIJ
Water Chestnuts	6.3 (0.5)	14	29	43	67	DEFGHIJ
Root Vegetable	6.3 (0.4)	10	38	48	79	DEFGHIJ
Peas	6.3 (0.3)	41	91	132	69	DEFGHIJ
Bean Sprouts	6.2 (0.5)	10	27	37	73	DEFGHIJ
Jicama	6.0 (0.4)	35	56	91	62	EFGHIJK
Asparagus	6.0 (0.3)	25	59	84	70	EFGHIJK
Edamame	5.9 (0.4)	21	39	60	65	FGHIJK
Cauliflower	5.9 (0.3)	39	78	117	67	FGHIJK
Okra	5.7 (0.7)	11	12	23	52	FGHIJK
Eggplant	5.7 (0.7)	10	13	23	57	FGHIJK
Bamboo Shoots	5.6 (0.6)	4	15	19	79	GHIJK
Squash	5.4 (0.3)	34	55	89	62	HIJK
Brussel Sprouts	5.3 (0.5)	28	33	61	54	IJK
Beets	4.9 (0.4)	28	34	62	55	JK
Onion	4.9 (0.3)	64	67	131	51	JK
Artichoke	4.6 (0.6)	13	12	25	48	K
Average	6.9 (0.05)					

^1^ Ratings were made on a scale from 1 = “Hate it” to 10 = “Love it”. ^2^ Unacceptable = mean liking rating < 5. ^3^ Acceptable = mean liking rating ≥ 5. ^4^ Percentage of children who found vegetable acceptable = Number of children who found vegetable acceptable/total number of children who ever tried the vegetable. ^5^ Vegetables with the same letter have mean liking ratings that are not statistically different.
